# No species-level losses of s2m suggests critical role in replication of SARS-related coronaviruses

**DOI:** 10.1038/s41598-021-95496-4

**Published:** 2021-08-09

**Authors:** Clément Gilbert, Torstein Tengs

**Affiliations:** 1 Université Paris-Saclay, CNRS, IRD, UMR Évolution, Génomes, Comportement et Écologie, 91198 Gif-sur-Yvette, France; 2 Section of Molecular Toxicology, Department of Environmental Health, Norwegian Institute of Public Health, Oslo, Norway

**Keywords:** Molecular evolution, Viral genetics

## Abstract

The genetic element s2m has been acquired through horizontal transfer by many distantly related viruses, including the SARS-related coronaviruses. Here we show that s2m is evolutionarily conserved in these viruses. Though several lineages of severe acute respiratory syndrome coronavirus 2 (SARS‑CoV‑2) devoid of the element can be found, these variants seem to have been short lived, indicating that they were less evolutionary fit than their s2m-containing counterparts. On a species-level, however, there do not appear to be any losses and this pattern strongly suggests that the s2m element is essential to virus replication in SARS-CoV-2 and related viruses. Further experiments are needed to elucidate the function of s2m.

The coding capacity of severe acute respiratory syndrome coronavirus 2 (SARS‑CoV‑2) has been investigated in great detail^1^, and the secondary structure of genomic RNA elements has also been studied^2,3^, but the biological significance of all of these components has not yet been fully elucidated. One of the annotated elements in the reference SARS-CoV-2 genome is the stem-loop II (s2m) element (Genbank accession NC_045512.2, position 29,728–29,768) that was originally described in astroviruses^4^. s2m is a 41-bp sequence located in the non-coding 3′ part of the SARS-CoV-2 genome. It has been found in members of a least four different virus families, including several lineages of coronaviruses^5,6^. There also seems to be a xenolog of s2m in some insect species, which likely results from endogenization of s2m-containing viral elements^7^. The evolutionary relationships between these homologs remain unclear, but it appears as if s2m has been horizontally transferred between distantly related organisms several times^6^. The function is unknown, but the high degree of conservation is consistent with this locus being under selective pressure.

Phylogenetic analyses support several acquisitions of s2m within the coronavirus family, with one gain basal to a cluster of SARS-related betacoronaviruses^5^. This cluster encompasses both SARS-CoV and SARS-CoV-2, as well as many related virus species, primarily isolated from bat species^7,8^. We have done a comprehensive phylogenetic analysis in order to map the distribution of s2m within the *Coronaviridae* subfamily (CoV). In particular, we have tried to assess whether there have been any losses of s2m within the clusters where this motif can be found, with emphasis on the SARS-related species.

All CoV nucleotide and amino acid sequence data were downloaded from GenBank. Based on an alignment of protein sequences from distantly related CoV species, two regions within the ORF1ab polyprotein were identified that could reliably be aligned across a broad range of accessions. The corresponding amino acid sequences from the reference SARS-CoV-2 genome (NC_045512.2 coding positions 10,334–13,468 and 13,462–21,552) were used as query sequences in tblastn sequence similarity searches against the CoV nucleotide data. When tabulating the results, the best matching sequence for every unique GenBank ‘ORGANISM’ identifier was extracted (Supplementary Table 1). In order to score a viral species as having s2m, the motif had to be found near the 3′ end of the genome with a maximum of one mismatch compared to published s2m sequences^4–7,9^ in at least one accession from the corresponding ‘ORGANISM’ identifier. To remove redundancy in the 436 CoV amino acid sequences that were retrieved from GenBank while retaining their full phylogenetic diversity, we aligned them using MAFTT^10^ and removed ambiguously aligned blocks with GBLOCKS^11^. We then used mothur^12^ to clusterize s2m-containing sequences and sequences devoid of s2m at 0.1% and 2.5% distance threshold, respectively. We chose to use a higher clustering threshold for sequence devoid of s2m because these sequences were not the focus of our study and were thus primarily included to place s2m-containing sequences in their evolutionary context. The resulting alignment of 133 amino acid sequences was subjected to a phylogenetic analysis using PHYML 3.0^13^ with the LG + G + I model, as determined by ProtTest 3^14^.

The resulting unrooted topology (Fig. 1) revealed three monophyletic clusters of s2m-containing operational taxonomic units (OTUs). The tree was highly supported, and in addition to two s2m-containing clades comprising isolates stemming from birds, a large group of SARS-related s2m-containing OTUs could readily be identified. This cluster included sequences sampled from several different bat species in addition to eight other vertebrates (Fig. 1). The most basal member of this cluster was Bat Hp-betacoronavirus Zhejiang2013, the only member thus far described from the *Betacoronavirus* subgenus *Hibecovirus*^15^. After excluding a small number of accessions without coverage in the 3′-end of the genome, this clade represented 183 unique ORGANISM identifiers (collapsed into 44 mothur-defined groups, see Supplementary Table 1) that all contained s2m.

**Figure 1:**
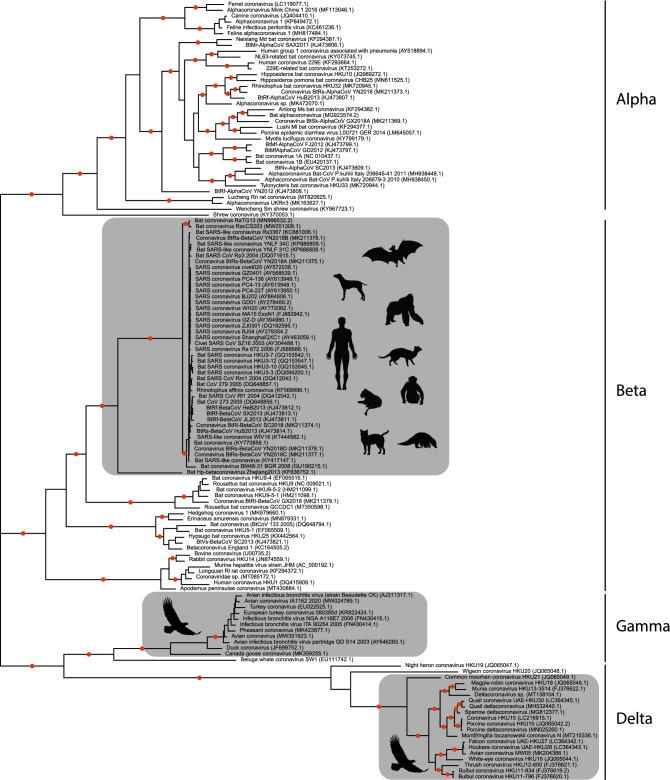
Unrooted maximum-likelihood tree of mothur-clusterized coronavirus ORF1ab sequences. Terminal edges represent single isolates or clusters of highly similar sequences represented by a random sequence within the cluster (see main text and Supplementary Table 1 for details). In addition to the data downloaded from GenBank, the analysis also included sequences from GISAID for strains isolated from lesser known hosts (i.e. cats, dogs, etc., see Supplementary Table 1 for details). Grey boxes represent s2m-containing accessions and *Coronaviridae* genera names are shown. Red dots indicate branches with 100% bootstrap support.

Though s2m showed no species-level losses within any of the three clusters, the vast amount of sequence data available from SARS-CoV-2 isolates permit a detailed analysis of how this motif might behave on a virus lineage-level. Sequence data and corresponding metadata from 537 360 SARS-CoV-2 isolates were downloaded from the GISAID database^16^. The 3′ end of high-quality genomes was screened for the presence of s2m single nucleotide polymorphisms (SNPs) and indels. A large number of SNP variants were observed, and, as expected, many of these correlated strongly with virus lineages (as defined by PANGOLIN annotation; Supplementary Table 2)^17^. Looking at indel variants, there also appeared to be lineage-specific variability and several isolates with complete deletion of s2m were observed (Fig. 2; Supplementary Table 3). Two lineages (B.1.1.311 and B.1.160.7) were found to be dominated by s2m deletion mutants (representing 63.7% and 76.1% of the submitted sequences, respectively). Lineage B.1.1.311 had complete deletion of the entire s2m region, whereas lineage B.1.160.7 had a smaller lesion (Fig. 2; DelSeq_1183 and DelSeq_325). Both lineages seem to have had peak distribution Fall 2020 and to have emerged within the United Kingdom (https://cov-lineages.org/lineages/lineage_B.1.1.311.html and https://cov-lineages.org/lineages/lineage_B.1.160.7.html). These lineages have obviously been viable, but their subsequent decline could imply that they were less fit than other emerging strains. Phylogenetic analyses of lineages containing s2m deletion mutants indicated that the primary genetic lesion often is the deletion of a small section of s2m, followed by complete elimination of the element from the lineage’s genome (data not shown).

**Figure 2:**
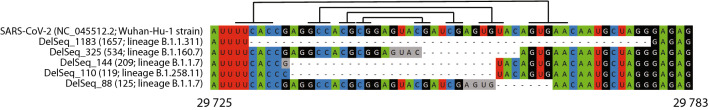
Alignment of the five most common SARS-CoV-2 s2m indel mutants in the GISAID database. Brackets indicate stem-forming regions as described for SARS-CoV^9^ and the Wuhan-Hu-1 strain has been included as a reference (nucleotide positions below indicate Wuhan-Hu-1 genome coordinates). Number of instances in the GISAID database and dominant lineage have been indicated (parenthesis after sequence name) and grey boxes show nucleotide(s) positions where there are multiple equally parsimonious ways of making the alignment.

Coronaviruses have been studied extensively in order to identify regions under selective pressure^18^. A recent study identified s2m as having the highest mutation rate in the SARS-CoV-2 genome and the authors suggest that this could be interpreted as either loss of purifying constraints or the result of diversifying selection^19^. Some early reports also proposed that s2m could be involved in recombination events^20^. It is reasonable to assume that the function of s2m is tightly linked with the element’s secondary structure. Assuming that the structure is not dependent on interactions with factors that have yet to be identified, an analysis of the canonical SARS-CoV-2 genome using an in vivo-based approach indicated that the structure of s2m deviates significantly from the structure observed for SARS-CoV^3^. The two versions of s2m differ in two positions, constituting two transversions that both seem to disrupt the stem-forming ability of s2m^3^. It is thus unclear if s2m in SARS-CoV and SARS-CoV-2 are functionally equivalent.

In our opinion, the fact that this element never seems to be lost at the species level within the SARS-related coronaviruses suggests that s2m became essential to virus replication after being acquired through horizontal transfer. Both cellular genes and non-coding RNAs acquired by double-stranded DNA viruses through horizontal transfer have been shown to become fixed in viral species, most likely due to their positive effect on viral replication^21–24^. On the contrary, populations of the AcMNPV baculovirus continuously receive transposable elements (TE) from their moth hosts, but all TE copies integrated into the viral genomes become rapidly lost, probably because they impose a fitness cost to the virus^25,26^.We argue that for s2m to be non-essential for viral replication, its distribution within the SARS-related coronaviruses should be significantly more patchy, due to frequent losses.

Though the exact function of s2m has not yet been revealed, several hypotheses have been entertained, including interference with the translational machinery of infected cells^9^, involvement in gene regulation through RNA silencing^6^ and protection of genomic/transcriptomic virus RNA from ribonuclease degradation^5^. The effect of swapping the 3′ UTR regions between s2m-containg and s2m-deficient virus strains has also been investigated, but the effect on viral fitness seems subtle^27^. Due to s2m’s highly conserved nature, it has also been suggested as a potential drug target^28,29^ and as a polymerase chain reaction (PCR) primer site for exploring virus diversity^30^. Further studies are needed in order to elucidate the role of s2m in virus replication, not just within the coronaviruses, but in all virus families where this horizontally transferred element has been detected.

## Supplementary Information

Supplementary Tables.
